# Supplementary material for: The Polygenic Risk Score Knowledge Base offers a centralized online repository for calculating and contextualizing polygenic risk scores
Source: Commun Biol. 2022 Sep 2;5:899. doi: 10.1038/s42003-022-03795-x (PMC9438378; doi:10.1038/s42003-022-03795-x)
Supplement: Supplementary file 2 — Supplementary Information [file 42003_2022_3795_MOESM2_ESM.pdf]

Supplementary Information: The Polygenic Risk Score Knowledge Base offers a centralized online repository for calculating and contextualizing polygenic risk scores

Madeline L. Page[1,*], Elizabeth L. Vance[1,*], Matthew E. Cloward[2,*], Ed Ringger[2], Louisa Dayton[2], Mark T.W. Ebbert[1,3,4], for the Alzheimer's Disease Neuroimaging Initiative[**], Justin B. Miller[1,3,5,+], John S.K. Kauwe[2,+]

[1] Sanders-Brown Center on Aging, University of Kentucky, Lexington, KY 40504, USA

[2] Department of Biology, Brigham Young University, Provo, UT 84602, USA

[3] Division of Biomedical Informatics, Department of Internal Medicine, University of Kentucky, Lexington, KY 40506, USA

[4] Department of Neuroscience, University of Kentucky, Lexington, KY 40506, USA

[5] Department of Pathology and Laboratory Medicine, University of Kentucky, Lexington, KY 40506, USA

Correspondence should be addressed to JSKK. Tel: 801-422-2993; Email: kauwe@byu.edu

[*] These authors contributed equally.

[+] These authors jointly supervised this project.

** Data used in preparation of this article were obtained from the Alzheimer's Disease Neuroimaging Initiative (ADNI) database (adni.loni.usc.edu). As such, the investigators within the ADNI contributed to the design and implementation of ADNI and/or provided data but did not participate in analysis or writing of this report. A complete listing of ADNI investigators can be found at: http://adni.loni.usc.edu/wp-content/uploads/how_to_apply/ADNI_Acknowledgement_List.pdf

Table of Contents

Supplementary Tables *3*

Supplementary Table 1: PRSKB database associations_table **3**

Supplementary Table 2: PRSKB database study_table **4**

Supplementary Table 3: PRSKB database linkage disequilibrium clumps **5**

Supplementary Table 4: Demographics of ADNI participants **5**

Supplementary Table 5: Phenotypic variance in ADNI explained by the PRSKB and PRSice-2 ...... **6**

Supplementary Figures *7*

Supplementary Figure 1: PRSKB data compilation pipeline **7**

Supplementary Figure 2: Linkage disequilibrium clumping **8**

Supplementary Figure 3: PRSKB calculator interface **9**

Supplementary Figure 4: Sample JSON output **10**

Supplementary Figure 5: GWA study browser interface **11**

Supplementary Figure 6: PRSKB command-line interface usage instructions **12**

Supplementary Figure 7: PRSKB command-line interface menu **12**

Supplementary Figure 8: Severe Covid-19 with respiratory failure polygenic risk score distribution for UK Biobank individuals **13**

Supplementary Figure 9: ADNI polygenic risk scores using GWA summary statistics from Lambert et al., 2013 ...... **14**

Supplementary Figure 10: ADNI polygenic risk scores using GWA summary statistics from Lo et al., 2019 ...... **15**

Supplementary Figure 11: PRSice-2 comparison: Lambert et al., 2013 ...... **16**

Supplementary Figure 12: PRSice-2 comparison: Lo et al., 2019 ...... **17**

Supplementary Figure 13: PRSice-2 comparison: Jansen et al., 2019 ...... **18**

Supplementary Note *19*

Supplementary Note 1: Linkage disequilibrium clumping **19**

Supplementary References *20*

Supplementary Tables

Supplementary Table 1: PRSKB database associations_table

Column Name	Description
**id**	Primary key column
**snp**	Reference SNP ID number
**hg38**	Location of the snp in the GRCh38 assembly
**hg19**	Location of the snp in the GRCh37 assembly
**hg18**	Location of the snp in the NCBI36 assembly
**hg17**	Location of the snp in the NCBI35 assembly
**trait**	Trait associatied with the snp
**gene**	Gene(s) reported with the snp
**raf**	Risk allele frequency
**riskAllele**	The allele associated with the odds ratio
**pValue**	The P-value for the SNP risk allele
**pValueAnnotation**	GWA study catalog of the P-value
**oddsRatio**	Reported odds ratio
**lowerCI**	Lower value for the 95% confidence interval
**upperCI**	Upper value for the 95% confidence interval
**betaValue**	Reported beta value
**betaUnit**	Unit in which beta value is reported
**betaAnnotation**	GWA study catalog of the beta value
**ogValueTypes**	Original value type (beta or odds ratio) used in the GWA study
**sex**	Sex associated with the P-value
**numAssociationsFiltered**	Number of associations filtered for this study
**citation**	First author's name and the year the study was published
**studyID**	The GWA study catalog accession ID for the study from which the association was identified

Data in this table is downloaded from the NHGRI-EBI GWAS Catalog 1 and filtered to only include associations that contain both an odds ratio and risk allele value and reside on an autosomal chromosome. Additionally, only non-haplotype associations are preserved.

Supplementary Table 2: PRSKB database study_table

Column Name	Description
**studyID**	GWA Study Catalog accession ID
**pubMedID**	PubMed ID for the GWA study
**trait**	Trait as reported by the GWA study catalog
**reportedTrait**	Trait as reported by the study authors
**citation**	First author's name and the year the study was published.
**altmetricScore**	Altmetric score of the GWA study—used to reflect study impact
**ethnicity**	Ethnicity of the study samples
**superPopulation**	1000 Genomes super population in which study samples reside
**initialSampleSize**	Size of initial study sample
**replicationSampleSize**	Size of replication study sample
**sex**	Sex of the study samples
**pValueAnnotation**	P-value annotation description
**betaAnnotation**	Beta value annotation description
**ogValueTypes**	Original value type (beta or odds ratio) used in the GWA study
**numAssociationsFiltered**	Number of associations filtered for this study
**title**	Title of the study
**lastUpdated**	Date the last update for this table took place

Data in this table is downloaded from the NHGRI-EBI GWAS Catalog [1] and describes the studies from which the associations in *study_association_table* were identified.

Supplementary Table 3: PRSKB database linkage disequilibrium clumps

Column Name	Description
**snp**	Reference SNP ID number
**position**	Chromosome number and base position of the snp for this specific reference assembly
**african_clump**	Linkage disequilibrium clump identifier for African population
**american_clump**	Linkage disequilibrium clump identifier for American population
**eastAsian_clump**	Linkage disequilibrium clump identifier for East Asian population
**european_clump**	Linkage disequilibrium clump identifier for European population
**southAsian_clump**	Linkage disequilibrium clump identifier for South Asian population

There is a separate clumps table for each combination of reference genome: *hg38_clumps, hg19_clumps, hg18_clumps*, and *hg17_clumps*. The variants in this table are downloaded from 1000 Genomes [2]. The clump identifier number allows us to quickly determine which variants belong to the same linkage disequilibrium region.

Supplementary Table 4: Demographics of ADNI participants

	Alzheimer's Disease (CDR≥1.0)	Mild Cognitive Impairment (CDR=0.5)	Cognitive Normal (CDR=0.0)
**Number of Subjects**	592	98	118
**Female Count (%)**	245 (41.39%)	56 (57.14%)	61 (51.70%)
**Average Age**	73.28±7.35	73.03±7.04	73.17±5.59

Supplementary Table 5: Phenotypic variance in ADNI explained by the PRSKB and PRSice-2

Group 1	Group 2	Study Authors	Study ID	Trait	Statistical Test	PRSKB Test Statistic	PRSKB P-value	PRSice-2 Test Statistic	PRSice-2 P-value
CDR=1	CDR≤0.5	Lambert, et al.	GCST002245	Alzheimer's disease	Mann Whitney U Test	68160	0.002295184	39451	0.002863105
CDR=1	CDR≤0.5	Jansen, et al.	GCST007320	Alzheimer's disease	Mann Whitney U Test	71392	2.60584E-05	35914	1.07586E-05
CDR=1	CDR≤0.5	Lo, et al.	GCST009496	Alzheimer's Disease	Mann Whitney U Test	74035	2.59068E-07	36471	1.1125E-07
CDR≥0.5	CDR=0	Lambert, et al.	GCST002245	Alzheimer's disease	Welch's Two-Sample T-Test	1.74402877	0.083138866	1.895648761	0.059849022
CDR≥0.5	CDR=0	Jansen, et al.	GCST007320	Alzheimer's disease	Mann Whitney U Test	45651	0.000338969	27829	0.000252806
CDR≥0.5	CDR=0	Lo, et al.	GCST009496	Alzheimer's disease	Mann Whitney U Test	48483	1.18525E-06	27211.5	6.29065E-07

Supplementary Figures

Supplementary Figure 1: PRSKB data compilation pipeline

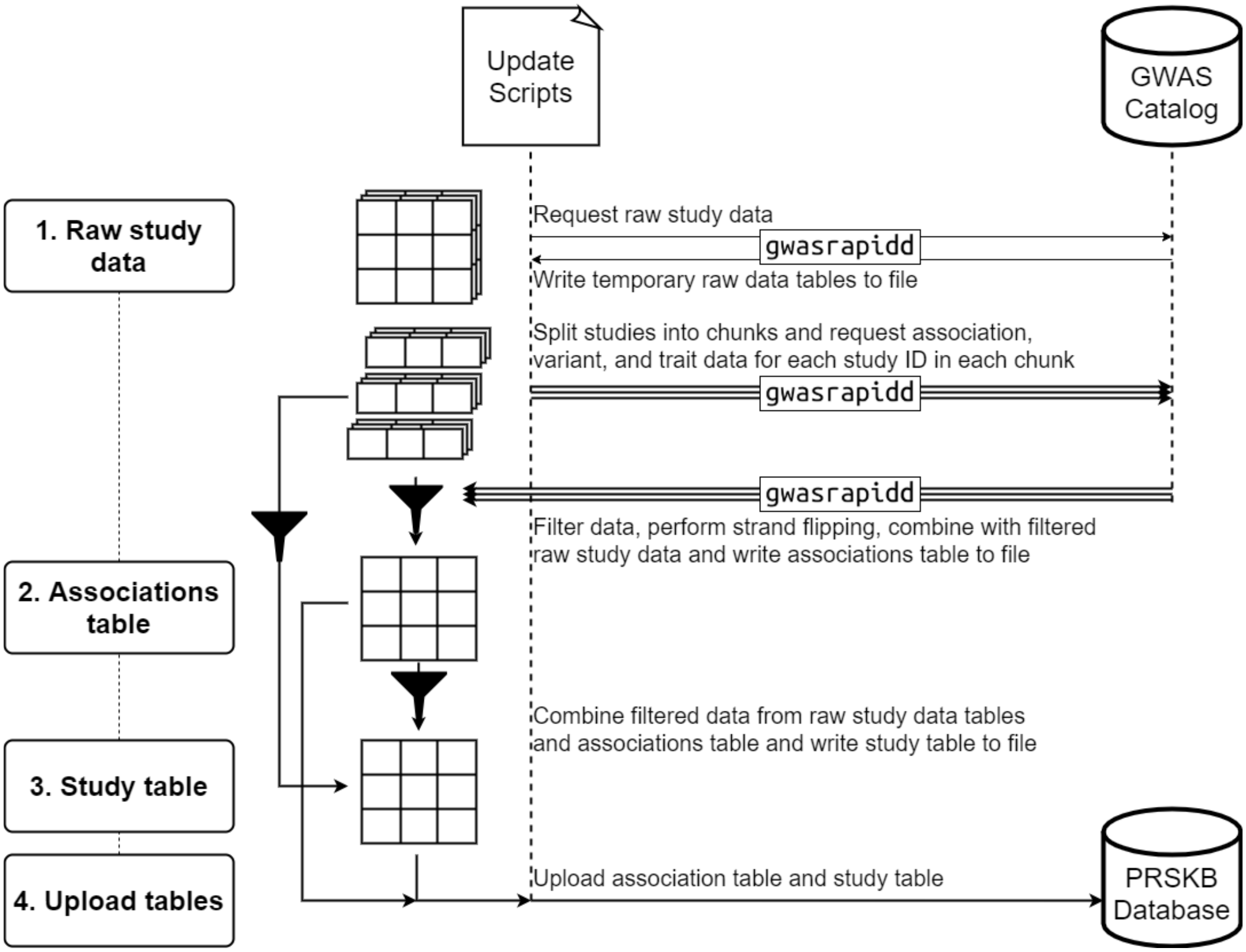

Every month, the PRSKB database is automatically synced with the NHGRI-EBI GWAS Catalog using a collection of shell, R, and Python scripts. Data from the GWAS Catalog is downloaded via the gwasrapidd R library, which uses the GWAS Catalog's API to connect to the database. All filtering and sorting of data downloaded
is done by the PRSKB's scripts. Arrows in this diagram represent the flow of data.
1. The scripts first download raw GWA study data from the GWAS Catalog using the gwasrapidd R library. The data are then written out to temporary TSV tables.
2. Association data are downloaded for each study ID in the temporary raw data tables. The study IDs are split into groups to speed up association data download time. The association data are filtered so that only non-haplotype autosomal associations that have risk alleles and beta values (or odds ratios) are preserved. The filtered data undergoes strand flipping, in which alleles reported on the opposite strand are corrected. The data is formatted and written to the associations_table.tsv 3. Data from the associations table and the temporary TSV tables are filtered, formatted, and sorted and written to the study_table.tsv.
4. Finally, the associations_table.tsv and the study_table.tsv are uploaded to the PRSKB database.
.

Supplementary Figure 2: Linkage disequilibrium clumping

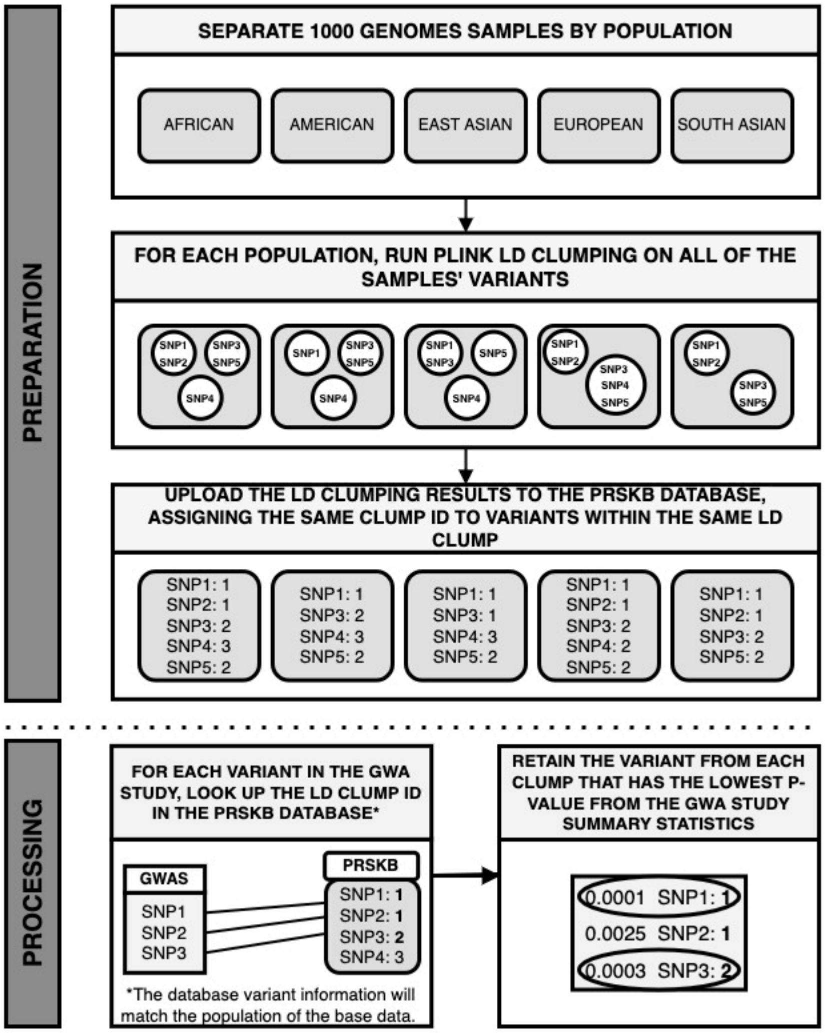

Supplementary Figure S1: Linkage disequilibrium clumping. The LD clumping procedure used by the PRSKB consists of two main parts: 1. Preparation and 2. Processing.

The preparation was performed once, in conjunction with the building of the PRSKB tool. First, we separated the 1000 Genomes samples by population. Then, for each population, we ran PLINK[2] LD Clumping on each of the samples' variants. Generally, PLINK LD Clumping is used to select a single variant with the most significant association with a given trait from each LD region. However, because our intent was to create general LD regions without regard to any specific trait, we assigned each 1000 Genomes variant a p-value of 0 in order to produce groups of unordered variants. Based on previous polygenic risk score analyses that use the clumping method, we used an r-squared threshold of 0.25 and a kb threshold of 500[3,4]. After applying this process for both the hg19 and hg38 available reference genome data, we converted the variant coordinates in each LD clump to the hg17 and hg18 reference genomes. Next, we assigned the same LD clump ID number to variants within the same LD region and uploaded this information to the PRSKB database. Linkage disequilibrium (LD) clumping files are stored on our server and are available online by using the population and reference genome. The URL for the European population (EUR) using reference genome hg38 would be written as follows: https://prs.byu.edu/get_clumps_download_file?refGen=hg38&superPop=EUR.

The processing step occurs each time a PRS calculation is performed. First, for each variant in the GWA study in question, the calculator queries the database to retrieve the corresponding LD clump ID based on the population of the GWA study's samples and the user-specified reference genome. Then, for each sample in the query data, the calculator retains the variant from each LD region that has the lowest p-value in the GWA study summary statistics. This final set of retained variants is then used to calculate a risk score for that sample.

Supplementary Figure 3: PRSKB calculator interface

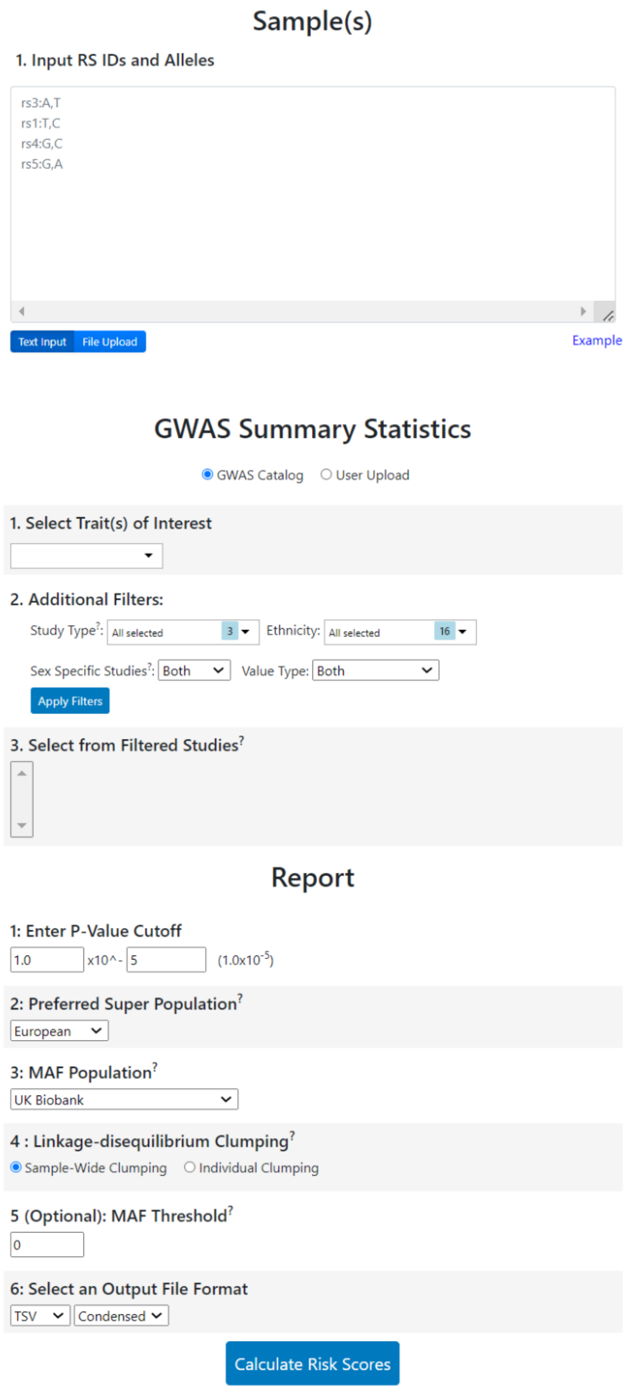

Supplementary Figure 3: PRSKB calculator interface. The PRSKB calculator is accessed under the "Calculate" tab at https://prs.byu.edu. Users run the PRSKB on their browser using a text or VCF file with genotype information. Users select the studies used for risk score calculations, indicate the p-value threshold for variants to include in the risk score calculations, and select the output file format (condensed TSV, verbose TSV, or JSON).

Supplementary Figure 4: Sample JSON output


```
[{
    "studyID": "GCST004365",
    "reportedTrait": "Blood Protein Levels",
    "trait": "[Pyruvate Dehydrogenase Acetyl-Transferring] Kinase Isozyme 1, Mitochondrial Measurement",
    "citation": "Suhre et al. 2017",
    "pValueAnnotation": "(ea, [pyruvate dehydrogenase (acetyl-transferring)] kinase isozyme 1, mitochondrial)",
    "betaAnnotation": "NA",
    "scoreType": "beta",
    "units (if applicable)": "unit",
    "snpsExcludedDueToCutoffs": 0,
    "usedSuperPop": "EUR",
    "samples": [
        {
            "sample": "SAMP001",
            "polygenicRiskScore": "-0.2859",
            "percentile": "7-42",
            "snpOverlap": 1,
            "includedSnps": 1,
            "protectiveAlleles": "rs12052479",
            "riskAlleles": "",
            "variantsWithoutRiskAllele": "rs12052479",
            "variantsInHighLD": ""
        },
        {
            "sample": "SAMP002",
            "polygenicRiskScore": "NF",
            "percentile": "NA",
            "snpOverlap": 1,
            "includedSnps": 1,
            "protectiveAlleles": "",
            "riskAlleles": "",
            "variantsWithoutRiskAllele": "rs12052479",
            "variantsInHighLD": ""
        },
        {
            "sample": "SAMP003",
            "polygenicRiskScore": "-0.5718",
            "percentile": "0-6",
            "snpOverlap": 1,
            "includedSnps": 1,
            "protectiveAlleles": "rs12052479",
            "riskAlleles": "",
            "variantsWithoutRiskAllele": "",
            "variantsInHighLD": ""
        }
    ]
},{
    "studyID": "GCST004643",
    "reportedTrait": "1,5-Anhydroglucitol Levels",
    "trait": "1,5 Anhydroglucitol Measurement",
```


Supplementary Figure 4: Sample JSON output.

Supplementary Figure 5: GWA study browser interface

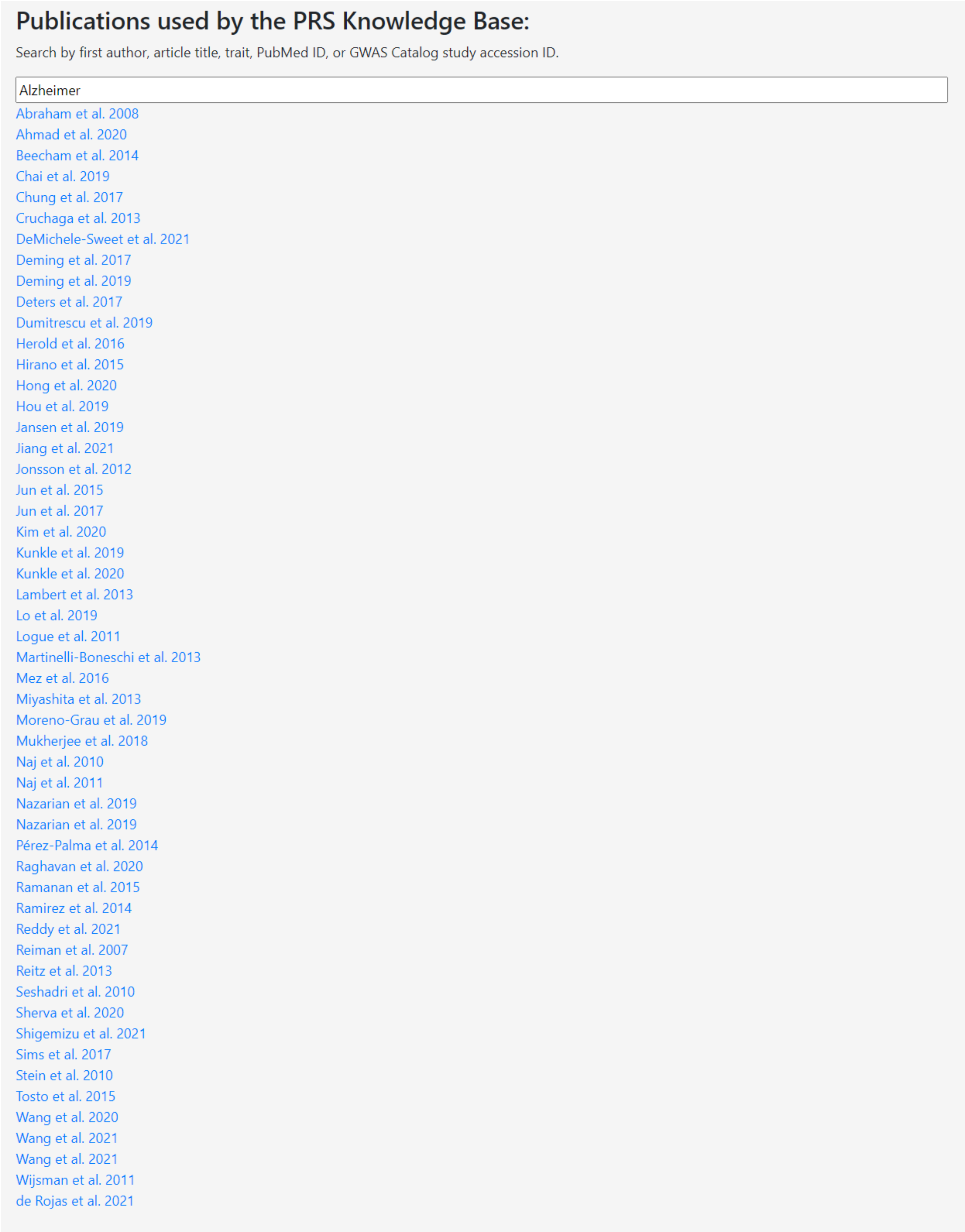

Supplementary Figure 5: GWA study browser. The GWA study browser can be found under the "Studies" tab at prs.byu.edu or at "Option 2: Search for a specific study or trait" on the command-line interface menu.

Supplementary Figure 6: PRSKB command-line interface usage instructions

```
USAGE:

./runPrsCLI.sh -f [VCF file path OR rsIDs:genotype file path] -o [output file path (tsv or json format)] -c [p-value cutoff (ex: 0.05)] -r [refGen {hg
17, hg18, hg19, hg38}] -p [preferred GWA study super population {AFR, AMR, EAS, EUR, SAS}]

Optional parameters to filter studies:
   -t traitList ex. -t acne -t insomnia -t "Alzheimer's disease"
   -k studyType ex. -k HI -k LC -k O (High Impact, Large Cohort, Other studies)
   -i studyIDs ex. -i GCST000727 -i GCST009496
   -e ethnicity ex. -e European -e "East Asian"
   -y value type ex. -y beta -y "Odds Ratio"
   -g sex in study ex. -g male -g female -g exclude
Additional Optional parameters:
   -v verbose ex. -v (indicates a more detailed TSV result file. By default, JSON output will already be verbose.)
   -s stepNumber ex. -s 1 or -s 2
   -n number of subprocesses ex. -n 2 (By default, the calculations will be run on all available subprocesses)
   -u path to GWAS data to use for calculations. Data in file MUST be tab separated and include the correct columns (see 'Learn about user supplied GW
AS data for calculations' or the CLI readme)
   -a reference genome used in the GWAS data file
   -b indicates that the user supplied GWAS data uses beta coefficent values instead of odds ratios
   -q sets the minor allele frequency cohort to be used (also is the cohort used for reporting percentiles) ex. -q adni-ad (see the menu to learn more
 about the cohorts available)
   -m omits reporting percentiles
   -x sets the cutoff minor allele frequency value
   -l individual-specific LD clumping ex. -l
   -h imputation threshold ex. -h 0.5
```

Supplementary Figure 6: PRSKB command-line interface usage instructions. The usage instructions are accessed by running the runPrsCli.sh bash script without any of the parameters.

Supplementary Figure 7: PRSKB command-line interface menu

```
==============================================================================
                   PRSKB Command-Line Menu/Instructions
==============================================================================
Welcome to the PRSKB command-line menu. Here you can learn about the different
parameters required to run a polygenic risk score (PRS) calculation, search
for a specific study or diesease, display available ethnicities for filtering,
view usage, or run the PRSKB calculator.

Select an option below by entering the corresponding number
then pressing [Enter].

 _ _ _ _ _ _ _ _ _ _ _ _ _ _ _ _ _ _ _ _ _ _ _ _ _ _ _ _ _ _
|                                                              |
| Options Menu                                                 |
| 1 - Learn about Parameters                                   |
| 2 - Search for a specific study or trait                     |
| 3 - View available ethnicities for filter                    |
| 4 - View usage                                               |
| 5 - Learn about user supplied GWAS data for calculations     |
| 6 - Run the PRSKB calculator                                 |
| 7 - Quit                                                     |
 _ _ _ _ _ _ _ _ _ _ _ _ _ _ _ _ _ _ _ _ _ _ _ _ _ _ _ _ _ _
#?
```

Supplementary Figure 7: PRSKB command-line interface menu. The CLI menu can be accessed through running the runPrsCLI.sh bash script without any of the parameters.

Supplementary Figure 8: Severe Covid-19 with respiratory failure polygenic risk score distribution for UK Biobank individuals

Ellinghaus et al. 2020

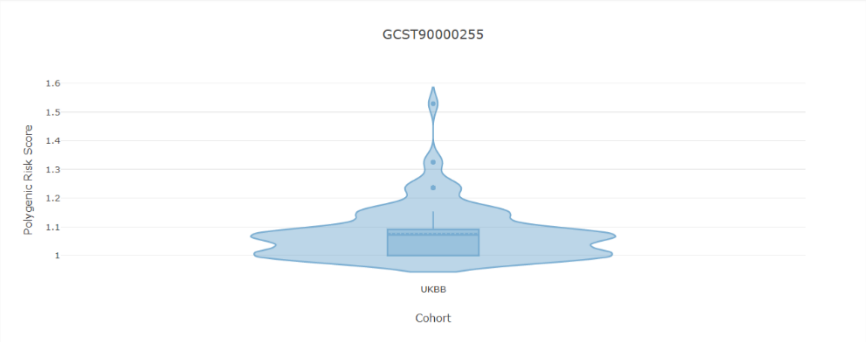

Violin Box Line

Summary Values	UKBB
Used Super Population	EUR
Min	1
Max	1.52853
Median	1.07187
Range	0.528529
Mean	1.0742
Geometric Mean	1.07142
Harmonic Mean	1.0687932234838582
Standard Deviation	0.0795569
Geometric Standard Deviation	1.0735784963805666

Title: Genomewide Association Study of Severe Covid-19 with Respiratory Failure.

Citation: Ellinghaus et al. 2020

Disease/Trait: Covid-19

Reported Trait: Severe Covid-19 Infection With Respiratory Failure (Analysis I)

P-value Annotation: NA

Beta Annotation: NA

Value Type: OR

Pubmed ID: 32558485

Altmetric Score: 6652.614

SNPs Used: ^
UKBB SNPs Used: rs11385942, rs657152

Supplementary Figure 8: Severe Covid-19 with respiratory failure polygenic risk score distribution for UK Biobank individuals, based on GWA summary statistics reported by Ellinghaus, et al.[1]*. This is an example of the type of graphics and data that are available under the "Visualize" tab at prs.byu.edu. Users can look at the distributions of risk scores for any study using the UK Biobank, 1000 Genomes, or ADNI datasets.*

Supplementary Figure 9: ADNI polygenic risk scores using GWA summary statistics from Lambert et al., 2013

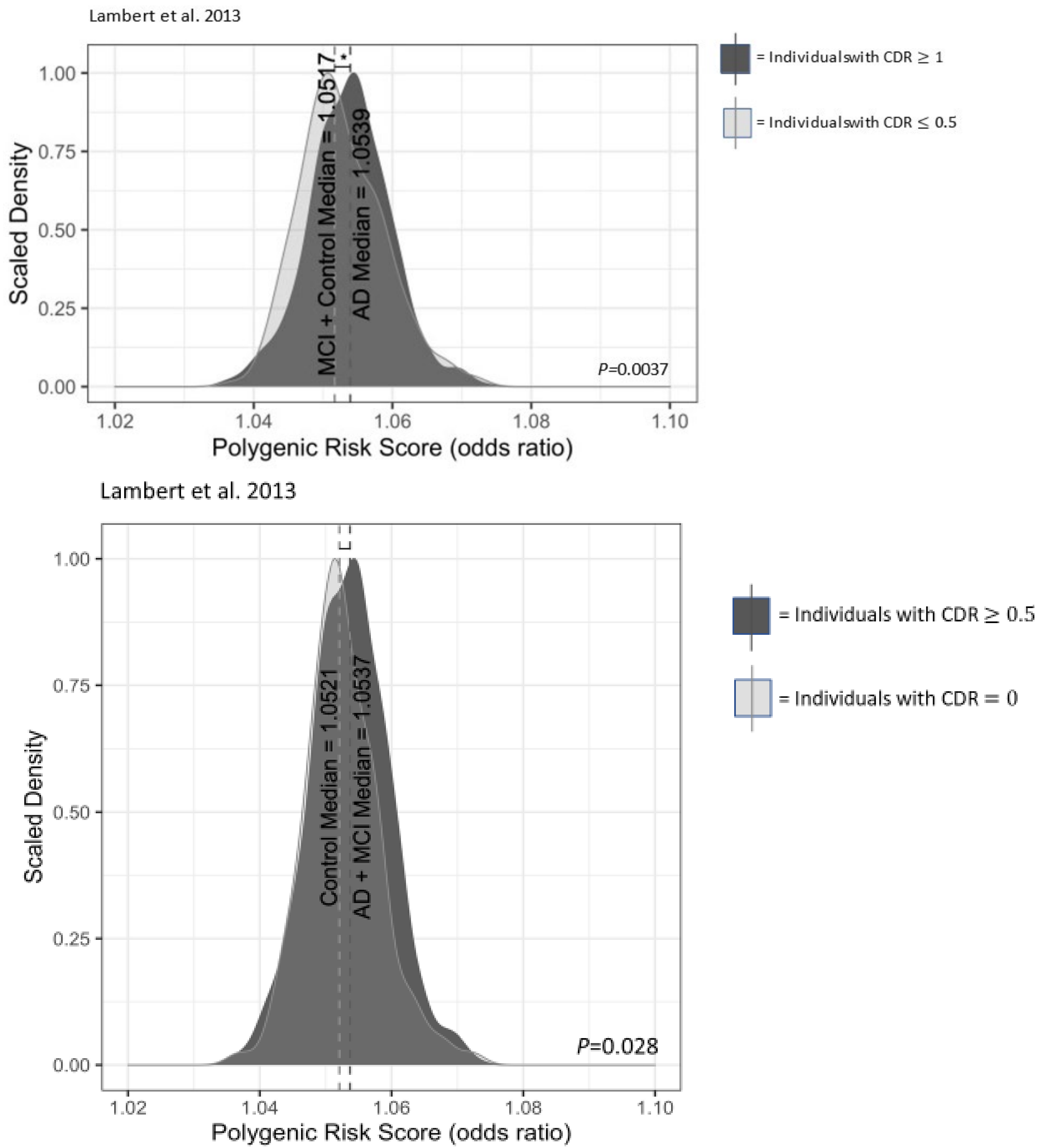

Supplementary Figure 9: Top: Polygenic risk scores for Alzheimer's disease for individuals with clinical Alzheimer's disease (CDR≥1) versus all other individuals. Bottom: Polygenic risk scores for Alzheimer's disease for individuals with clinical dementia (CDR ≥0.5) versus individuals with normal cognition.

Supplementary Figure 10: ADNI polygenic risk scores using GWA summary statistics from Lo et al., 2019

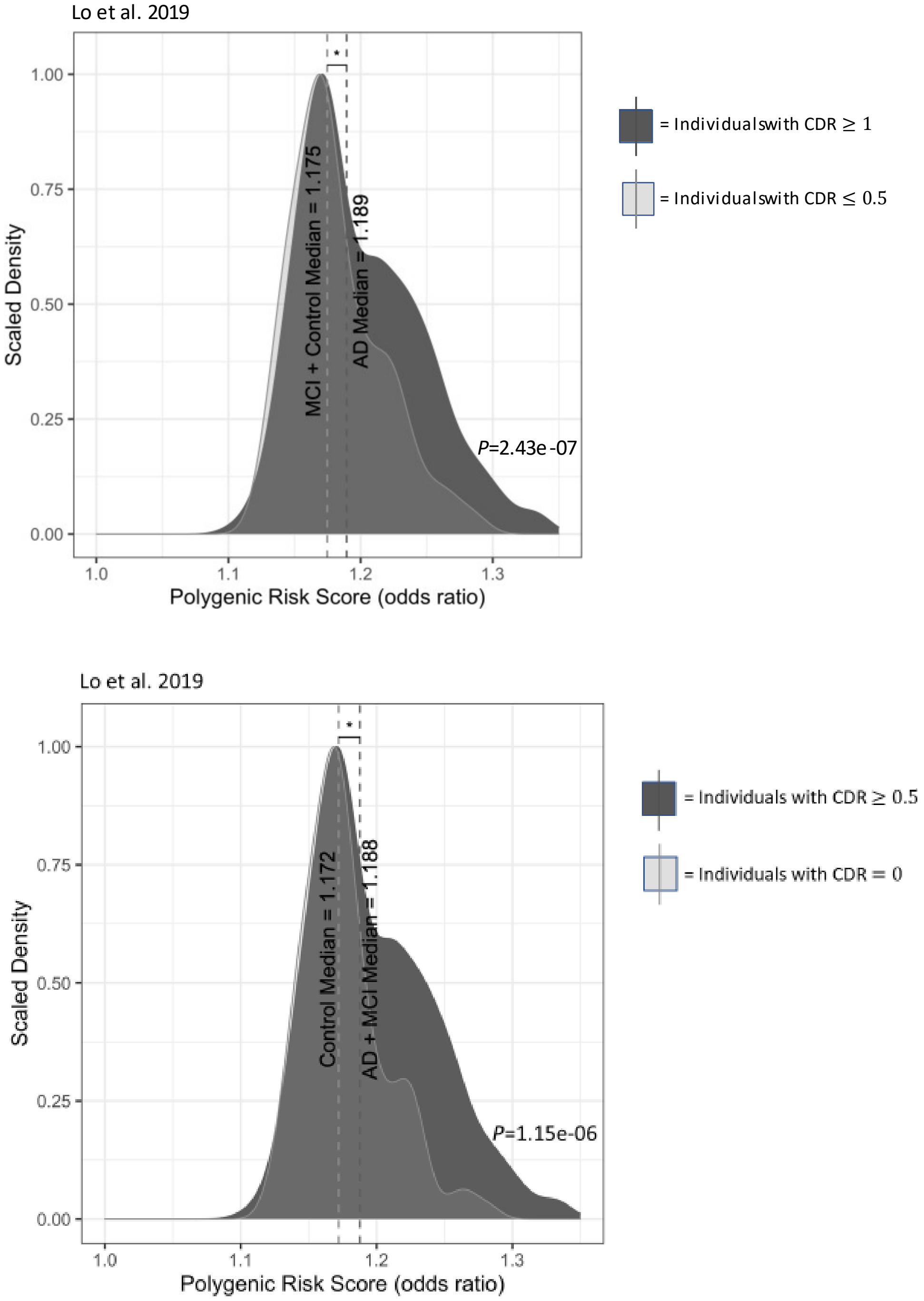

Supplementary Figure 10: Top: Polygenic risk scores for Alzheimer's disease for individuals with clinical Alzheimer's disease (CDR≥1) versus all other individuals. Bottom: Polygenic risk scores for Alzheimer's disease for individuals with clinical dementia (CDR ≥0.5) versus individuals with normal cognition.

Supplementary Figure 11: PRSice-2 comparison: Lambert et al., 2013

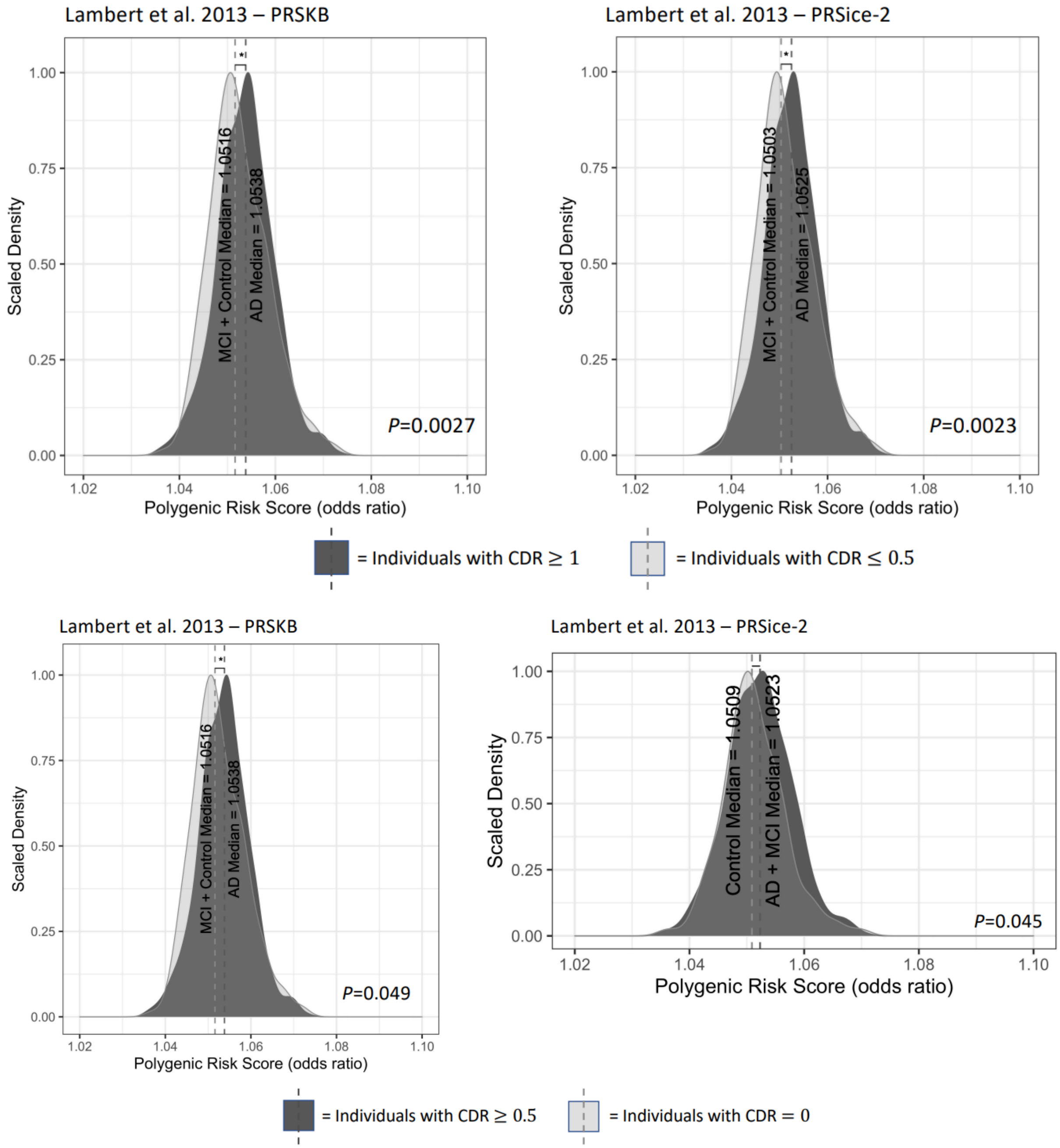

Supplementary Figure 11: Comparison of polygenic risk scores calculated by the PRSKB and PRSice-2. PRSice-2 reports polygenic risk scores that center on 0, so 1.0 was added to each PRSice-2 score to put it on the same scale as the PRSKB, which centers polygenic risk scores based on odds ratios around 1.0. In ADNI, r6656401 reports G and AAG as alternative alleles. The AAG variant was removed for this comparison to facilitate direct comparisons between the two tools because PRSice-2 does not allow multiple risk alleles to occur at the same genomic position. Top: Polygenic risk scores for Alzheimer's disease for individuals with clinical Alzheimer's disease (CDR≥1) versus all other individuals. Bottom: Polygenic risk scores for Alzheimer's disease for individuals with clinical dementia (CDR ≥0.5) versus individuals with normal cognition.

Supplementary Figure 12: PRSice-2 comparison: Lo et al., 2019

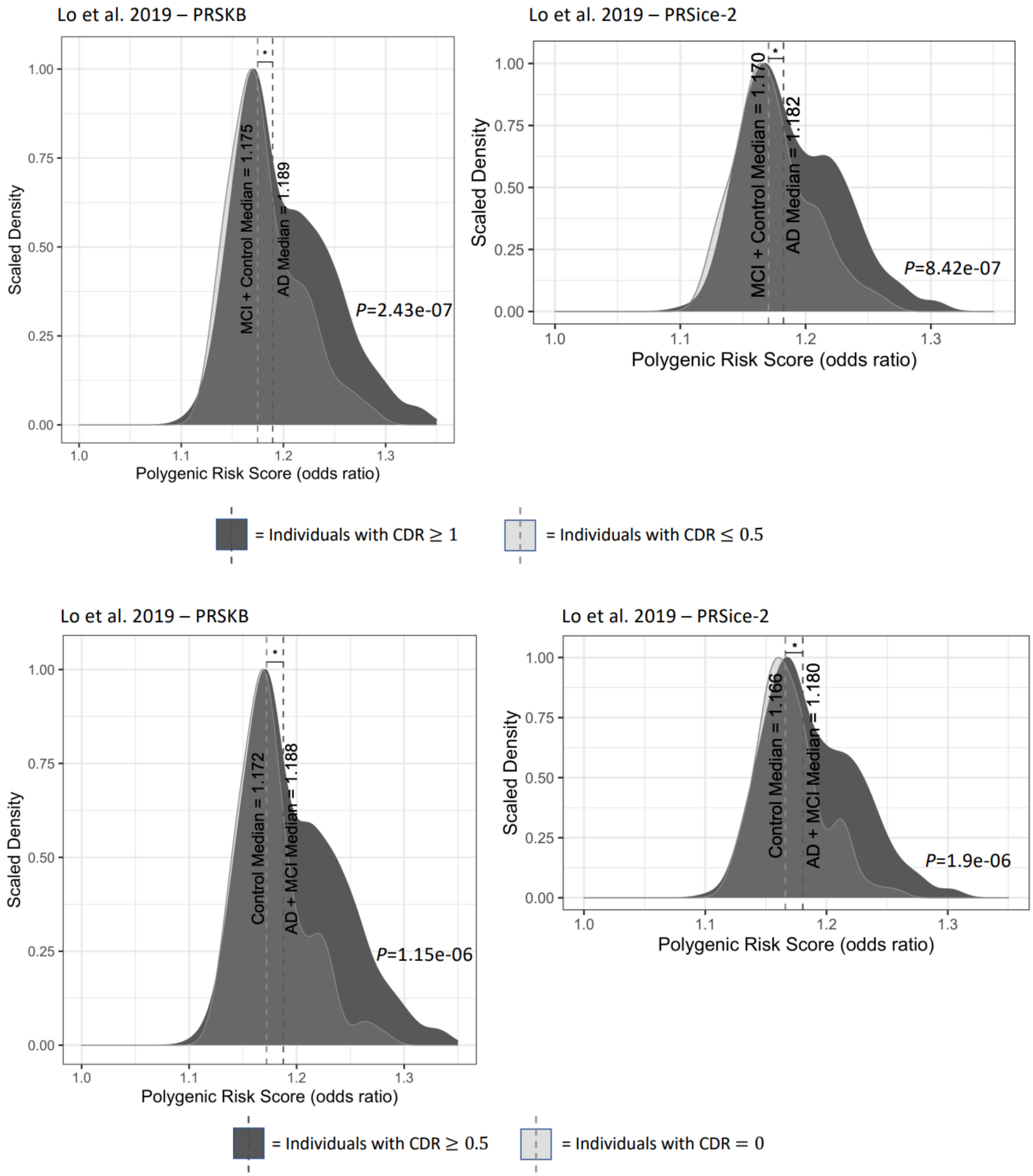

Supplementary Figure 12: Comparison of polygenic risk scores calculated by the PRSKB and PRSice-2. PRSice-2 reports polygenic risk scores that center on 0, so 1.0 was added to each PRSice-2 score to put it on the same scale as the PRSKB, which centers polygenic risk scores based on odds ratios around 1.0. Top: Polygenic risk scores for Alzheimer's disease for individuals with clinical Alzheimer's disease (CDR≥1) versus all other individuals. Bottom: Polygenic risk scores for Alzheimer's disease for individuals with clinical dementia (CDR ≥0.5) versus individuals with normal cognition.

Supplementary Figure 13: PRSice-2 comparison: Jansen et al., 2019

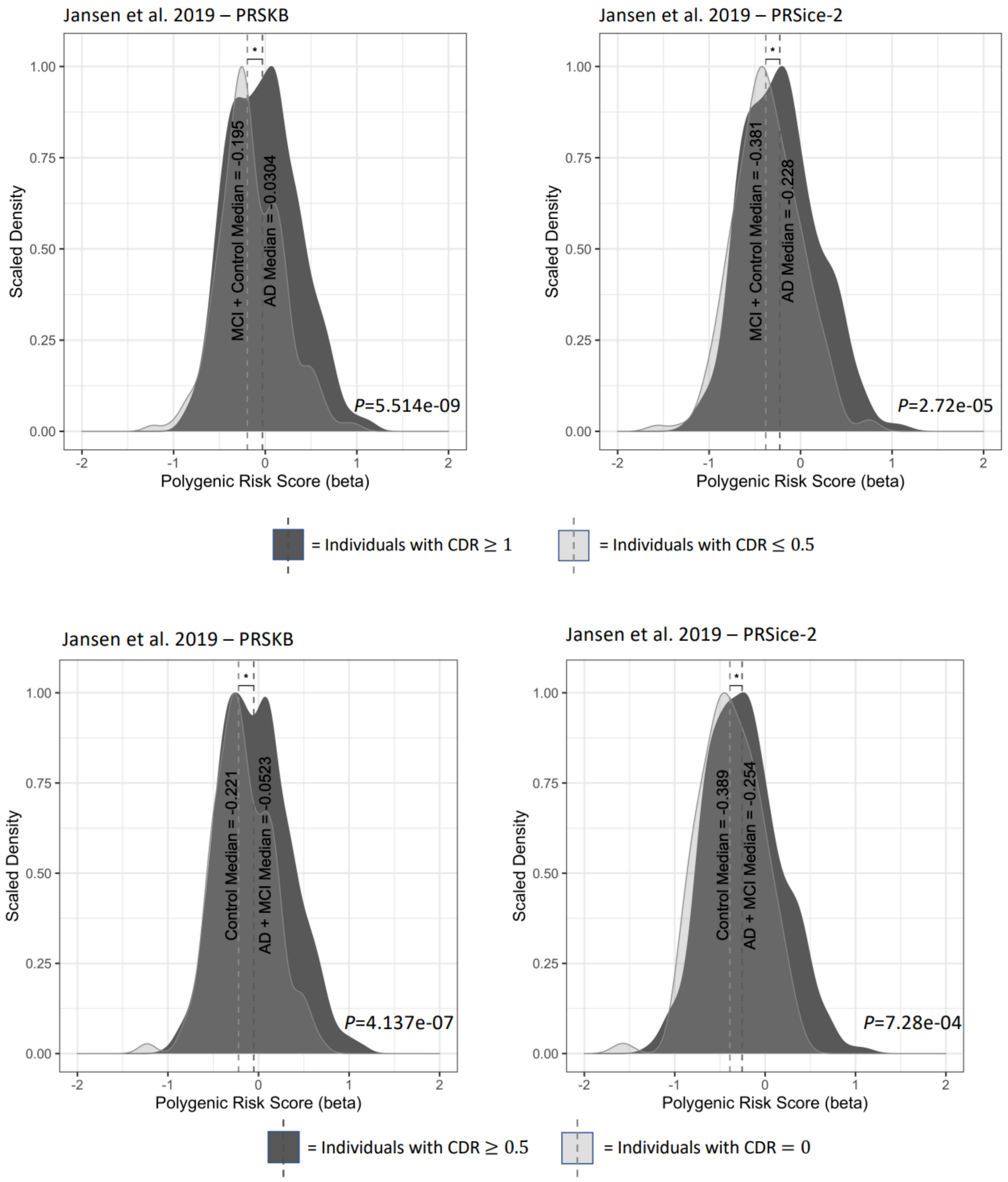

Supplementary Figure 13: Comparison of polygenic risk scores calculated by the PRSKB and PRSice-2. Since the PRSKB centers polygenic risk scores based on beta values around 0.0, no transformations were performed on these data, and the results are directly comparable. In ADNI, r6656401 reports G and AAG as alternative alleles. The AAG variant was removed for this comparison to facilitate direct comparisons between the two tools because PRSice-2 does not allow multiple risk alleles to occur at the same genomic position. We Top: Polygenic risk scores for Alzheimer's disease for individuals with clinical Alzheimer's disease (CDR≥1) versus all other individuals. Bottom: Polygenic risk scores for Alzheimer's disease for individuals with clinical dementia (CDR ≥0.5) versus individuals with normal cognition.

Supplementary Note

Supplementary Note 1: Linkage disequilibrium clumping

Linkage disequilibrium (LD) clumping files are stored on our server and are available online by using the population and reference genome. The URL for the European population (EUR) using reference genome hg38 would be written as follows:

https://prs.byu.edu/get_clumps_download_file?refGen=hg38&superPop=EUR

In order to account for LD in the PRS calculations, we pre-computed the LD regions for each variant in the 1000 Genomes database. We used the PLINK[2] LD Clumping command, which requires reference genotype data in order to calculate LD between the variants present in a target input file.

We use 1000 Genomes data for both the reference and target files. To create the reference files, we downloaded 1000 Genomes variant call format (VCF) files and separated each file by super population (African, American, East Asian, European, and South Asian). Next, we used PLINK to convert each VCF file into a binary file set by running the following command, where ${population} is one of the population specific VCF files.

PLINK --vcf ${population} --make-bed

The variants intended to be grouped into LD regions are found in the ${variant_file}. The PLINK --clump operation scans the ${variant_file} and extracts fields with the headers 'SNP' (reference SNP ID number) and 'P' (p-value). For each population, we created a variant file, where the 'SNP' column contained the variants listed in the corresponding population-filtered VCF file. The 'P' column typically contains p-values for the association that each SNP has with a designated trait. This value is used to arrange the variants within each clump based on their association with the given trait. Since our intent was to identify LD regions for all variants from 1000 Genomes, regardless of their association with a certain trait, we filled every value in this column with '0'. As a result, the final LD clumps are unordered.

For each super population (African, American, East Asian, European, and South Asian), we executed the LD clumping command as follows, where ${reference_data} refers to the population-specific binary file set and ${variant_file} indicates the file with the list of rsIDs.

PLINK --bfile ${reference_data} --clump ${variant_file} --clump-p1 1 --clump-p2 1 --clump—r2 0.25 --clump-kb 500

The following four parameters determined the level of clumping that was performed:
--clump-p1: The significance threshold for the variant with the lowest p-value in a clump
--clump-p2: The significance threshold for all other variants in a clump
--clump-r2: The r-squared value that signifies the linkage disequilibrium threshold for clumping
--clump-kb The physical distance (kb) threshold for clumping

In order to maintain as many variants as possible in our final regions, we used a p-value threshold of 1 for both p1 and p2. Based on previous polygenic risk score analyses that use the clumping method, we assigned an r-squared threshold of 0.25 and a kb threshold of 500[3,4].

After applying this process for both the hg19 and hg38 available reference genome data, we converted the variant coordinates in each LD clump to the hg17 and hg18 reference genomes.

Supplementary References

1 Ellinghaus, D. *et al.* Genomewide Association Study of Severe Covid-19 with Respiratory Failure. *The New England journal of medicine* **383**, 1522-1534, doi:10.1056/NEJMoa2020283 (2020).

2 Purcell, S. *et al.* PLINK: a tool set for whole-genome association and population-based linkage analyses. *Am J Hum Genet* **81**, 559-575, doi:10.1086/519795 (2007).

3 Privé, F., Vilhjálmsson, B. J., Aschard, H. & Blum, M. G. B. Making the Most of Clumping and Thresholding for Polygenic Scores. *The American Journal of Human Genetics* **105**, 1213-1221, doi:https://doi.org/10.1016/j.ajhg.2019.11.001 (2019).

4 Wray, N. R. *et al.* Research review: Polygenic methods and their application to psychiatric traits. *Journal of child psychology and psychiatry, and allied disciplines* **55**, 1068-1087, doi:10.1111/jcpp.12295 (2014).
