# Supplementary material for: The Polygenic Risk Score Knowledge Base offers a centralized online repository for calculating and contextualizing polygenic risk scores
Source: Commun Biol. 2022 Sep 2;5:899. doi: 10.1038/s42003-022-03795-x (PMC9438378; doi:10.1038/s42003-022-03795-x)
Supplement: Supplementary file 3 — Description of Additional Supplementary Files [file 42003_2022_3795_MOESM3_ESM.pdf]

Description of Additional Supplementary Files

File name: Supplementary Data 1
Description: Genetic variants used in calculating polygenic risk score from Lambert et al., 2013 (GWAS catalog ID: GCST002245).

File name: Supplementary Data 2
Description: Genetic variants used in calculating polygenic risk score from Jansen et al., 2019 (GWAS catalog ID: GCST007320).

File name: Supplementary Data 3
Description: Genetic variants used in calculating polygenic risk score from Lo et al., 2019 (GWAS catalog ID: GCST009496).

File name: Supplementary Data 4
Description: A sample condensed TSV output file including one line per study with the headers shown in this file.

File name: Supplementary Data 5
Description: A sample verbose TSV output file including one line per study with the headers shown in this file.

File name: Supplementary Data 6
Description: Difference between polygenic risk score distributions in individuals with a CDR=1 (Alzheimer's disease) and individuals with a CDR=0.5 or CDR=0 (MCI + Controls).

File name: Supplementary Data 7
Description: Difference between polygenic risk score distributions in individuals with a CDR=1 or CDR=0.5 (Alzheimer's disease + MCI) and individuals with a CDR=0 (Controls).
