# Supplementary material for: The Polygenic Risk Score Knowledge Base offers a centralized online repository for calculating and contextualizing polygenic risk scores
Source: Commun Biol. 2022 Sep 2;5:899. doi: 10.1038/s42003-022-03795-x (PMC9438378; doi:10.1038/s42003-022-03795-x)
Supplement: Supplementary file 11 — Reporting Summary [file 42003_2022_3795_MOESM11_ESM.pdf]

nature portfolio

Corresponding author(s): John S.K. Kauwe
Last updated by author(s): May 10, 2022

Reporting Summary

Nature Portfolio wishes to improve the reproducibility of the work that we publish. This form provides structure for consistency and transparency in reporting. For further information on Nature Portfolio policies, see our Editorial Policies and the Editorial Policy Checklist.

Statistics

For all statistical analyses, confirm that the following items are present in the figure legend, table legend, main text, or Methods section.

n/a	Confirmed	
☐	☒	The exact sample size (*n*) for each experimental group/condition, given as a discrete number and unit of measurement
☐	☒	A statement on whether measurements were taken from distinct samples or whether the same sample was measured repeatedly
☐	☒	The statistical test(s) used AND whether they are one- or two-sided<br>*Only common tests should be described solely by name; describe more complex techniques in the Methods section.*
☐	☒	A description of all covariates tested
☐	☒	A description of any assumptions or corrections, such as tests of normality and adjustment for multiple comparisons
☐	☒	A full description of the statistical parameters including central tendency (e.g. means) or other basic estimates (e.g. regression coefficient) AND variation (e.g. standard deviation) or associated estimates of uncertainty (e.g. confidence intervals)
☐	☒	For null hypothesis testing, the test statistic (e.g. *F*, *t*, *r*) with confidence intervals, effect sizes, degrees of freedom and *P* value noted<br>*Give P values as exact values whenever suitable.*
☒	☐	For Bayesian analysis, information on the choice of priors and Markov chain Monte Carlo settings
☐	☒	For hierarchical and complex designs, identification of the appropriate level for tests and full reporting of outcomes
☐	☒	Estimates of effect sizes (e.g. Cohen's *d*, Pearson's *r*), indicating how they were calculated

Our web collection on statistics for biologists contains articles on many of the points above.

Software and code

Policy information about availability of computer code

Data collection	The PRSKB integrates with the National Human Genome Research Institute-European Bioinformatics Institute (NHGRI-EBI) GWAS Catalog60 to provide the most up-to-date and comprehensive list of GWA studies. The GWAS Catalog is a publicly available database of GWA study summary statistics that allows individual research labs to submit full summary statistic files. The PRSKB automatically downloads, prunes, and reformats study and association data from the GWAS Catalog using the gwasrapidd R library61. The data are filtered to include only associations that contain both a beta value (or odds ratio) and the respective risk allele. Each variant is analyzed independently (i.e., risk haplotypes are excluded). Sex-specific variants are not included in the database. Finally, any allele that has been reported on the reverse strand is automatically detected and flipped to the forward strand. The strand-flipping procedure entails comparing each reported risk allele to the list of possible alleles for the specified variant from dbSNP62. If the reported risk allele does not exist in the list of possible alleles, the complement of the risk allele is checked against the dbSNP list. If the complement is present, then it is used as the reported risk allele for polygenic risk score calculations, as recommended by Choi, et al. 21.
Data analysis	Code Availability Statement:<br>All programs and code for this manuscript are publicly available at https://github.com/kauwelab/PolyRiskScore.<br><br>PRSKB Tool Structure<br>The PRSKB is divided into three key parts: the database, the server, and the client, as shown in Figure 1. More information on how the database was compiled is shown in Figure S1. The GWA study data, linkage disequilibrium clumping data, and association data are housed in a MySQL database on the PRSKB server. Tables S1-S3 expound on the information found in each database table. The variant associations from each study/trait combination are contained within a single associations table, which includes detailed summary statistics for each variant (see Table S1). The study table (see Table S2) contains detailed descriptions of each GWA study. Finally, there are four clumps tables, hg38 clumps, hg19 clumps, hg18 clumps, and hg17 clumps, that include linkage disequilibrium region identification numbers for variants in each of the five super populations from the 1000 Genomes project (see Table S3). The associations and study tables are automatically updated monthly with new associations added to the GWAS Catalog. The scripts for loading tables into the database are publicly available at https://github.com/kauwelab/PolyRiskScore/tree/master/update_database_scripts.

The server houses the application programming interface (API) endpoints for the PRSKB, running NodeJS using PM2 (https://pm2.keymetrics.io/) and NGINX (https://www.nginx.com/). While the user does not interact directly with the API endpoints, the client calls endpoints to download requested data needed to calculate polygenic risk scores. All calculations occur client-side to reduce strain on the server.

Users have two platforms from which they can calculate polygenic risk scores. The first platform is a web interface accessible at https://prs.byu.edu via a web browser that allows users to perform client-side calculations where user data are never uploaded to the PRSKB server. The second platform is a command-line interface (CLI) tool that can be run from the Linux or Mac command-line or from a bash shell on Windows. The CLI includes a bash script and four Python scripts. We recommend using the CLI to calculate polygenic risk scores for multi-sample VCF files, calculating scores spanning more than 50 GWA studies, and if the user prefers more control over their bioinformatics pipelines.

For manuscripts utilizing custom algorithms or software that are central to the research but not yet described in published literature, software must be made available to editors and reviewers. We strongly encourage code deposition in a community repository (e.g. GitHub). See the Nature Portfolio guidelines for submitting code & software for further information.

Data

Policy information about availability of data

All manuscripts must include a data availability statement. This statement should provide the following information, where applicable:

- Accession codes, unique identifiers, or web links for publicly available datasets
- A description of any restrictions on data availability
- For clinical datasets or third party data, please ensure that the statement adheres to our policy

This project is documented online at https://polyriskscore.readthedocs.io/en/latest/. A web interface is publicly available at https://prs.byu.edu/.

Field-specific reporting

Please select the one below that is the best fit for your research. If you are not sure, read the appropriate sections before making your selection.

☒ Life sciences ☐ Behavioural & social sciences ☐ Ecological, evolutionary & environmental sciences

For a reference copy of the document with all sections, see nature.com/documents/nr-reporting-summary-flat.pdf

Life sciences study design

All studies must disclose on these points even when the disclosure is negative.

Sample size

As of March 16, 2022, the PRSKB contains the following data that can be used for user-specific calculations of polygenic risk scores and contextualization against larger cohorts: 250,134 variant associations; 125,433 unique single nucleotide polymorphisms; 20,798 unique study and trait combinations; 10,366 GWA study identifiers; and 3,463 PubMed identifiers.

Data exclusions

The data are filtered to include only associations that contain both a beta value (or odds ratio) and the respective risk allele.

Various filters allow users to choose specific studies, populations, or study types (e.g., users can choose to include only studies with the highest Altmetric score77 or the largest study cohort reported by the GWAS Catalog, measured as the initial sample size plus the replication sample size).

Replication

We computed Alzheimer's disease polygenic risk scores and interactive graphics for the Alzheimer's Disease Neuroimaging Initiative (ADNI) database (adni.loni.usc.edu) to verify the efficacy of the PRSKB calculations.

Similar comparisons between Alzheimer's disease and cognitive normal controls in the ADNI dataset using GWA studies from Lambert, et al. 3, Jansen, et al. 2, and Lo, et al. 74 show that the PRSKB and PRSice-2 produce very similar distributions.

Randomization

GWAS were chosen from the GWAS Catalog. We rely on the summary statistics reported by previous studies. Randomization was not necessary because we used all available studies and all available participants in each cohort, who already had data labels (e.g., case/control).

Blinding

Our analyses did not include data collection, and blinding was not possible for the analyses. Since our manuscript describes a novel method, blinding should not be a factor in the outcome of these results.

Reporting for specific materials, systems and methods

We require information from authors about some types of materials, experimental systems and methods used in many studies. Here, indicate whether each material, system or method listed is relevant to your study. If you are not sure if a list item applies to your research, read the appropriate section before selecting a response.

Materials & experimental systems

n/a	Involved in the study
☒	☐ Antibodies
☒	☐ Eukaryotic cell lines
☒	☐ Palaeontology and archaeology
☒	☐ Animals and other organisms
☒	☐ Human research participants
☒	☐ Clinical data
☒	☐ Dual use research of concern

Methods

n/a	Involved in the study
☒	☐ ChIP-seq
☒	☐ Flow cytometry
☒	☐ MRI-based neuroimaging
